# Short- and long-term effects of tactile massage on salivary cortisol concentrations in Parkinson’s disease: a randomised controlled pilot study

**DOI:** 10.1186/1472-6882-13-357

**Published:** 2013-12-13

**Authors:** Carl-Johan Törnhage, Örjan Skogar, Astrid Borg, Birgitta Larsson, Laila Robertsson, Lena Andersson, Lena Andersson, Paulina Backström, Per-Arne Fall, Gunnar Hallgren, Birgitta Bringer, Miriam Carlsson, Ulla Birgitta Lennartsson, Håkan Sandbjörk, Johan Lökk

**Affiliations:** 1 Department of Pediatrics, Skaraborg Hospital, SE-541 85 Skovde, Sweden; 2 Department of Geriatrics, Ryhov Hospital, Jönkoping, Sweden; 3 Institution of Neurobiology, Care Sciences, and Society, Karolinska Institutet, Karolinska University Hospital Huddinge, Stockholm, Sweden; 4 Swedish Parkinson Foundation, Stockholm, Sweden; 5 Department of Neurology, Skaraborg Hospital, Skovde, Sweden; 6 Department of Geriatrics, University Hospital, Linkoping, Sweden

**Keywords:** Circadian rhythm, Complementary therapies, Cortisol, Massage, Parkinson disease, Stress

## Abstract

**Background:**

Parkinson’s disease (PD) is a chronic neurodegenerative disorder with limited knowledge about the normal function and effects of non-pharmacological therapies on the hypothalamic-pituitary-adrenal (HPA) axis. The aim of the study was to analyse the basal diurnal and total secretion of salivary cortisol in short- and long-term aspects of tactile massage (TM)_._

**Methods:**

Design: Prospective, Controlled and Randomised Multicentre Trial.

Setting and interventions: Forty-five women and men, aged 50–79 years, were recruited. Twenty-nine of them were blindly randomised to tactile massage (TM) and 16 of them to the control group, rest to music (RTM). Ten interventions were given during 8 weeks followed by a 26 weeks of follow up. Salivary cortisol was collected at 8 am, 1 pm, 8 pm, and 8 am the next day, on five occasions. With the first and eighth interventions, it was collected immediately before and after intervention.

Main outcome measures: The primary aim was to assess and compare cortisol concentrations before and immediately after intervention and also during the follow-up period. The secondary aim was to assess the impact of age, gender, body mass index (BMI), duration and severity of PD, effects of interventional time-point of the day, and levodopa doses on cortisol concentration.

**Results:**

The median cortisol concentrations for all participants were 16.0, 5.8, 2.8, and 14.0 nmol/L at baseline, later reproduced four times without significant differences. Cortisol concentrations decreased significantly after TM intervention but no change in diurnal salivary cortisol pattern was found. The findings of reduced salivary cortisol concentrations immediately after the interventions are in agreement with previous studies. However, there was no significant difference between the TM and control groups. There were no significant correlations between cortisol concentrations and age, gender, BMI, time-point for intervention, time interval between anti-parkinson pharmacy intake and sampling, levodopa doses, duration, or severity of PD.

**Conclusions:**

Diurnal salivary cortisol rhythm was normal. Salivary cortisol concentrations were significantly reduced after the TM intervention and after RTM, but there were no significant differences between the groups and no sustained long-term effect. No associations were seen between salivary cortisol concentration and clinical and/or pharmacological characteristics.

**Trial registration:**

ClinicalTrial.gov, NCT01734876 and FoU Sweden 108881.

## Background

### Why study the hypothalamic-pituitary-adrenal (HPA) axis in PD?

Parkinson’s disease (PD) is a chronic progressive neurodegenerative disorder accompanied by autonomic dysfunction and alterations in different regulatory mechanisms [1]. Typically, signs of PD are hypokinesia, rigidity, and tremor. Non-motor symptoms (NMS) such as mood changes, pain, and autonomic dysfunctions are frequent [2-4]. Many PD patients suffer from sleep disorders, apathy, tiredness, anorexia, and instability (hypotension). These symptoms can mimic reduced adrenal cortisol activity.

Health-related quality of life (HRQoL) is poor in people with PD compared with other disabled populations [5]. As the disease progresses, motor and non-motor complications become more severe, making patients’ adherence to medication even more important. A complicated dosing or titration schedule is a part of daily life in PD [6]. In addition to pharmacological treatment, there are non-pharmacological approaches aimed at alleviating the symptoms of PD. Complementary and alternative methods (CAM) are commonly used in PD patients [7]. Acute and chronic stress seem to raise the function of the hypothalamic-pituitary axis in terms of elevated concentrations of cortisol [8-10]. Several studies have shown salivary cortisol concentration to be an excellent mirror of hypothalamic-pituitary axis function [11], with fast reactions to changes in the surroundings [12]. We hypothesised that this would be a natural biomarker for stress in our study. In a previous study by our group, we found that median morning salivary cortisol concentrations and total cortisol secretion during the day were higher in PD than in an age- and sex-matched healthy reference group [8]. In a previous study by Hartmann [13], total serum cortisol was analysed every 15 min for 24 h. They found a higher diurnal cortisol secretion in PD compared with a healthy reference group. This study was very intensive, took place in a hospital situation, and did not analyse free biologically active cortisol. Could these results be a consequence of stress in the sampling situation?

Later studies have shown that free biologically active cortisol increases much more than total cortisol in situations of stress, intensive care, and so on [12]. Literature findings in 2003, when this study began, were sparse. There was no other similar study that had analysed diurnal cortisol secretion in PD during and after interventions and during a long follow-up period.

To confirm or reject the previous findings, we performed this study in a group of healthy patients with well-defined PD for a duration of more than 2 years. Their only medications were for PD and for chronic pain, and the disease was not so severe that they needed subthalamic deep brain stimulation, continuous release of levodopa in their duodenal bulb (Duo-Dopa), or apomorphine injections.

### What is the primary causal factor in HPA axis dysregulation: consequences of ageing, stress, or PD itself?

In a previous study, we compared the HPA axis function in PD patients with *and* without chronic pain [8]. Chronic pain was defined as significant *PD-related pain* more than three times/week during at least 3 months before inclusion. We compared salivary cortisol of PD patients with an age- and sex-matched healthy reference group. These analyses were performed identically and in the same laboratory. We found *higher* morning cortisol in both PD groups compared with the reference group. There was no significant difference between PD with and without chronic pain [8]. In a 76-year-old female, the diurnal cortisol curve was very prominent, with very high morning cortisol concentrations, in agreement with a well-functioning circadian rhythm.

In summary, the HPA axis seems to be up-gradated in PD patients without other disorders (somatic, psychiatric). The chronic pain (=stress) resulted in no difference. We found *no* correlation between salivary cortisol concentration (HPA axis function) and age, sex, BMI, adjusted daily dose of levodopa, or time interval between medication ingestion within 1 h either side of salivary sampling [8]. Therefore, PD itself seems to result in HPA axis dysregulation.

### Massage therapy

The Greek physician Hippocrates (460–377 B.C.) advocated rubbing as a treatment for stiffness and massage was the primary form of care for stiffness until the pharmaceutical revolution of the 1940s. Massage therapy has received empirical support for facilitating growth, reducing pain, increasing alertness, diminishing depression, and enhancing immune function [14].

In agreement with these historical experiences, massage therapists in our region have also experienced excellent effects on relaxation, reduced pain, increased motor function, and improved sleep in PD patients. Stress tolerance is low in this type of patient; HRQoL is known to be worse than in age- and sex-matched healthy persons as well as in patients with other chronic disorders such as stroke [15,16]. In an attempt to study the effects of this non-pharmacological approach on HPA axis function and some of the associated NMS such as pain and sleep disturbances, the Parkitouch study was initiated with the intention to report the effects of TM on salivary cortisol concentrations.

#### ***Main outcome measures***

The primary aim was to assess and compare cortisol concentrations before, during, and immediately after intervention with TM and RTM and also during the follow-up period. The secondary aim was to assess the impact of age, gender, body mass index (BMI), duration and severity of PD, effects of interventional time-point of the day, and levodopa doses on cortisol concentration.

## Methods

### Subjects

Patients with stable and well-defined PD for more than 2 years who fulfilled the established clinical criteria for diagnosis and with chronic pain were recruited from the outpatient departments of three medium-sized city hospitals in southern Sweden. Chronic pain was defined as the occurrence of PD-related pain for 3 days or more per week during at least 3 months prior to inclusion. Exclusion criteria were severe fluctuations in PD, concurrent existence of epilepsy, active malignancy, polyneuropathy, or other serious disease of somatic or psychiatric origin that could interfere with the study. Patients with severe abnormalities of blood parameters, electrolytes, liver or renal parameters such as bilirubin >20 mmol/L, creatinine >130 mmol/L, sedimentation rate >30 mm, or p/glucose > 6.7 mmol/L (fasting) were also excluded. Participation in other studies was not allowed.

### Pharmacological treatment

The patients had medication only for PD, and for chronic pain, mainly non-steroidal anti-inflammatory drugs (NSAID). We adjusted for the total levodopa equivalent dose for their dopamine agonists.

### Unified Parkinson Disease Related Scale (UPDRS)

UPDRS questionnaires I–IV were used to classify the participants into different groups in relation to cognition, mood, performance, motor function, activities of daily living, and adverse reactions to their medication [17].

### Procedures

To optimise our study design, salivary cortisol was sampled in the patients’ homes to minimise stress. This was done at exact time points according to detailed written instructions for sampling and storage of the samples in their refrigerator before sending the collected samples to the laboratory. All samples, five diurnal curves and six samples from two interventions (n = 26) from each person, were analysed at the same time point to minimise the intra- and inter-assay coefficients of variance (CV).

### Study design, recruitment, and randomisation

Participants were recruited from September 2004 to January 2009. The study was controlled and prospective, and the participants were randomised by computer to either tactile massage (TM) or the control group (RTM) by a lottery procedure. We had no adjustment with a block strategy in the randomisation procedure. All patients gave their written informed consent. An independent member of the Parkitouch Study Group was responsible for enrolment of all participants by communicating the blinded, computerised randomisation course. She informed the nurse by a telephone call. She took a prenumbered envelope from a box, opened the closed envelope, and informed about what type of individual intervention the participant should be given. Thereafter, she put the envelope back in the box.

**Figure 1:**
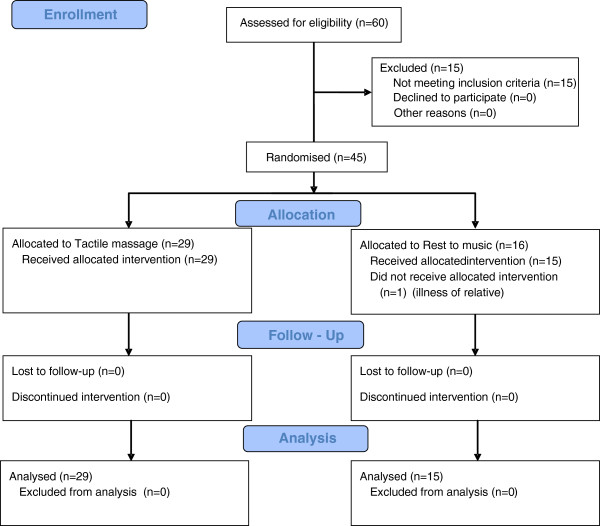
The CONSORT 2010 flow diagram.

For details about enrolment, intervention allocation, follow-up, and analysis see the CONSORT 2010 flow diagram (Figure 1) [18].

**Figure 2:**
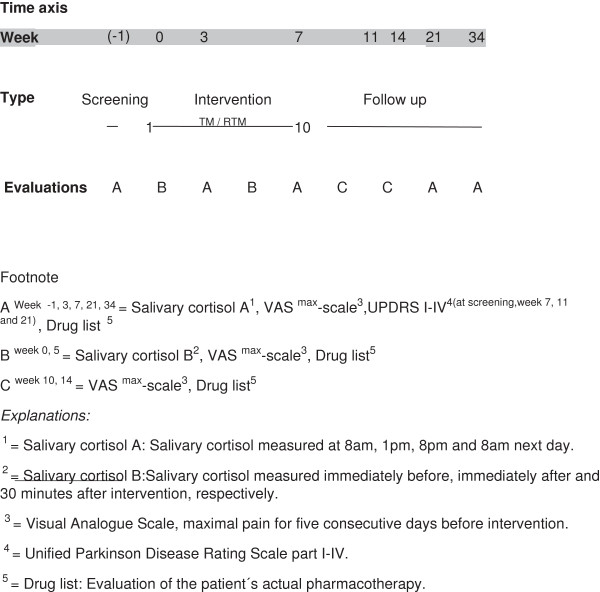
Time axis, interventions for Tactile massage and Rest to music groups.

Patients visited the outpatient clinic of the respective hospitals during the 34-week study period (Figure 2). Two interventions per week were performed during the first 3 weeks and thereafter, one intervention was performed per week. The 10th intervention was performed 8 weeks after randomisation, followed by a 26-week follow-up.

### Study intervention

All sessions were predetermined to be *performed during the period between 9 am and 12 am.* During TM, a specific oil was used, Fibro oil from Crearome AB, Gamleby, Sweden, mixed with Virgin oil comprising one-third of the total volume. TM was performed following detailed instructions, written in 2003 by licensed massage therapists and co-authors Laila Robertsson and Birgitta Larsson (see Additional file 1). They certified the competence of the participating local massage therapists. All individual TM interventions were given by the same therapist.

Patients randomised to RTM had the same external conditions. RTM was given in the same location as TM, with the same duration, and the other circumstances were identical to TM excluding the specific massage. The music was identical in both groups: Music for well-being II – Letting go of stress (LC6607 Fönix Musik, Sweden). The participants could regulate the sound level to a comfortable level.

### Collection of salivary cortisol samples

All time points for sampling were registered exactly in a protocol. For both groups, collection of salivary samples was done using a well-described technique [19]. In short, a cotton-based neutral swab was used; teeth should not be brushed and no food should be eaten within 30 min before sampling. Thereafter, a neutral swab was chewed for 2 min and later, the swab was placed in a plastic double lumen tube. Then, the tube was placed in a refrigerator at home until it was sent for further analysis within 3 days. In the lab, it was centrifuged at 3,000 rpm for 10 min at room temperature, followed by freezing at -20 to -80°C until assayed. All samples were analysed at the same time. The time-points for sampling were 8 am, 1 pm, 8 pm, and 8 am the next day and before, immediately after, and 30 min after intervention was finished. A commercial RIA-based technique for measurement of salivary cortisol was used (Spectria Cortisol I^125^ TM, Landskrona, Sweden).

### Measures

The total secretions of cortisol during the day, from 8 am–8 pm, and during the night, from 8 pm–8 am, were calculated using the formula for area under curve (AUC) from the zero level (AUC0 = AUCG), according to investigations of Preussner [20] and Fekedulegn [21]. All analyses of saliva from the same individual (n = 26) were performed simultaneously. Salivary cortisol during the day was measured at baseline, at 3, 8, 21, and 34 weeks after randomisation. In addition, the immediate effects (just before, immediately after, and 30 min after the intervention was finished) were measured at the first and the eighth interventions.

### Statistical analysis

STATISTICA version 8.0 and 10.0 (STATsoft Inc. Tulsa, OK, USA) and SPSS version 18.0 (SPSS Inc, Chicago, IL, USA) were used for the statistical evaluations. Non-parametric tests were used to adjust for the skewness in the subjects. Mann–Whitney U test and Kruskal-Wallis one-way analysis of variance (ANOVA) were used to compare the two groups. Friedman’s ANOVA was used to compare the diurnal cortisol rhythm and total cortisol secretion during the five study time-points. Wilcoxon’s matched-pairs signed-rank test was used to analyse individual diurnal rhythms and Spearman rank order correlation test was used to analyse the association between cortisol and clinical characteristics. All tests were two-sided and statistical significance was assumed at p < 0.05.

Power: In order to have a 20% difference in cortisol concentration (AUC) between groups, a total of 40 patients were needed to have 90% power with a significance level of 0.05.

#### ***Ethics***

The study was approved by the Ethics Committees at the University of Gothenburg (Ö 76203) and the University of Linkoping (D 03–673), Sweden.

## Results

Our study included 45 participants randomised to TM (n = 29) or RTM (n = 16). One participant in the RTM group was excluded immediately after randomisation because of disease in the family. There were no important harms or unintended effects that resulted in the discontinuation of intervention. Clinical and demographic characteristics at baseline were similar between groups (Table 1).

**Table 1:** Clinical and demographic characteristics of the two PD populations at baseline

**Group**	**Sex**	**Age**^**1**^	**Weight**^**2**^	**BMI**^**2**^	**H&Y **^**2,3**^	**UPDRS (I-IV) **^**2,4**^
“Tactile massage”	Male (n = 10)	50-78	86.5 (68.1/103.4)	26.6 (24.1/37.4)	1.5 (1.0/2.5)	31.5 (24.1/46.4)
	Female (n = 19)	60-79	64.7 (54.8/95.0)	25.0 (20.2/35.9)	2.5 (1.5/3.1)	39.0 (27.5/61.2)
“Rest to music”	Male (n = 6)	50-74	88.6 (62.0/102.0)	27.0 (23.6/31.5)	3.0 (1.5/3.0)	42.5 (32.0/57.0)
	Female (n = 9)	50-74	70.8 (44.5/92.4)	24.2 (17.8/31.2)	2.0 (1.0/4.0)	39.0 (21.0/78.0)

Values are given as range^1^ and medians/10^th^ and 90^th^ percentiles^2^. Hoehn and Yahr^3^, Unified Parkinson’s Disease Rating Scale^4^.

There were no statistical differences between the groups or gender. (Statistical method: Mann–Whitney U-test).

Follow-up rates were 100% and 93%, respectively. The natural diurnal negative slope of cortisol concentrations between 8 am and 1 pm was estimated to 2.0 nmol/h. Comparisons between TM and RTM groups at the time-points for awakening, sampling, interventions, and cortisol concentrations, delta cortisol, percentage changes of cortisol concentration, and total cortisol secretion during and within 30 min after intervention (AUC) are shown in Table 2.

**Table 2:** Baseline variables and cortisol concentrations at first and eighth intervention split by arms

	**RTM**	**(n = 14)**	**TM**	**(n = 28)**
**Age** (year)	62.5	(54–73)	66.0	(61–73)
**Gender** (m/f)	6/8		10/18	
**Time of wakening**				
First/eighth intervention	04.30-07.30		02.00-07.30	
	04.30-08.00		04.00-08.00	
**Time of sampling**				
First/eighth intervention	07.20-15.35		08.00-15.49	
	08.00-15.12		07.51-15.15	
**Time interval after wakening** (min)	90-585		110-543	
	55-578		**40**^**§**^-555	
**Cortisol** (nmol/L) (median/10/90%)				
Before first/eighth intervention	7.0	(4.5/23.9)	9.2	(4.1/17.0)
	7.2	(4.1/23.9)	7.5	(4.5/13.8)
After 0′	6.3	(2.6/18.7)	6.8	(2.5/15.4)
	6.6	(2.5/11.0)	5.7	(3.5/9.8)
After 30′	6.6	(2.4/8.3)	5.6	(2.6/13.2)
	4.8	(2.5/21.1)	4.6	(2.6/9.2)
Delta Cortisol (nmol/L)				
Before - after 0′				
First/eighth intervention	1.8	(-3.0/+5.5)	1.9	(-2.0/+6.2)
	2.6	(-2.5/+17.1)	1.7	(-2.0/+5.4)
Before- after 30′				
First/eighth intervention	2.4	(-0.3/+17.1)	3.4	(-2.2/+6.5)
	3.6	(-0.1/+13.2)	3.1	(-2.8/+7.5)
Percentage difference in Cortisol				
Before - after 0′				
First/eighth intervention	26.8	(-41.0/+42.4)	27.7	(-2.2/+55.4)
	38.8	(-38.5/+58.8)	26.1	(-30.9/+53.8)
Before- after 30′				
First/eighth intervention	45.8	(-4.5/+71.5)	33.3	(-12.9/+60.7)
	11.3	(-45.5/+29.2)	11.6	(-28.0/+43.7)
**Cortisol AUC**_**G**_				
Before - after 0′				
First/eighth intervention	350	(143/1271)	456	(189/954)
	338	(173/1030)	352	(221/656)
Before- after 30′				
First/eighth intervention	582	(255/1939)	662	(267/1366)
	562	(262/1614)	491	(303/854)

Footnote: TM = Tactile massage, RTM = Rest to music. AUC_G_ = area under curve from ground level.

Statistical methods used; Mann–Whitney-U, Chi-2-test, Median test and Kruskal-Wallis test. There were no statistical differences between groups. Eight time intervals < 60 min. at 8^th^ intervention and once at first intervention. ^**§**^This patient had concentrations, 7.7, 9.7, and 10.2 nmol/L, excluding a cortisol arousal reaction (CAR).

### Pharmacological treatment

No significant differences between the two groups were seen even when we integrated other forms of anti-PD drugs and recalculated the total dopaminergic load using formulas from the literature [22]. Forty-two of 44 patients were treated with levodopa with a median total dose of 625 mg/day after recalculation. The pharmacological treatment was essentially unchanged and only single extra doses of anti-PD treatment were taken during the study.

### Massage

The TM was performed for each individual patient by the same therapist for a mean duration of 52 minutes per session (range 40–79 minutes), with a total of 10 massages during a period of 8 weeks. At the first and eighth interventions, TM / RTM was given for 133 (10–293) and 109 (10–272) minutes, respectively, after intake of the morning PD medication.

All interventions were performed before 12 am, except in the case of three participants, from each type of intervention at first and eighth sessions. There was no statistical difference in salivary cortisol response between early and late interventions.

### Salivary cortisol

**Figure 3:**
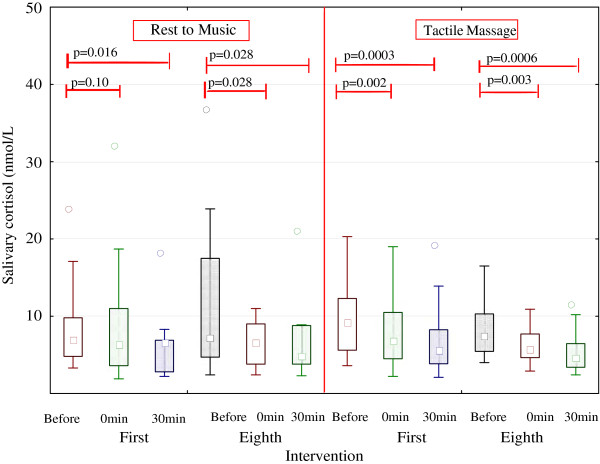
Short term effects of Tactile massage and Rest to music on salivary cortisol concentration.

Basal and short- and long-term effects of intervention on cortisol concentrations are shown in Figure 3 and Tables 2 and 3. At baseline, there was no significant difference between the groups. Salivary cortisol concentrations at baseline before the first intervention and at week 3, after the sixth intervention, were not significantly different between groups, as shown in Table 3. A comparison of the two groups regarding the total diurnal secretion of cortisol (AUC) showed no differences between groups (data not shown).

**Table 3:** Diurnal salivary cortisol concentration during intervention and follow up period

**WEEK**	**Tactile massage**				**Rest to music**				**p-values**^**1**^
	**8 am**	**1 pm**	**8 pm**	**8 am**	**8 am**	**1 pm**	**8 pm**	**8 am**	
**0**	14.3(5.8-28.9)	4.9(2.4-23.3)	2.8(1.6-7.1)	14.0(6.9-35.0)	18.5(3.9-28.5)	6.2(3.7-10.0)	2.8(1.1-8.5)	17.0(7.6-28.6)	ns
**3**	13.4(6.2-26.7)	6.3(3.1-10.1)	2.6(1.2-6.1)	14.1(6.7-29.5)	15.9(8.8-18.8)	3.9(3.0-10.5)	1.9(1.4-7.8)	12.4(8.1-34.0)	ns
**8**	12.1(6.6-37.3)	4.9(2.9-14.3)	2.5(1.5-4.8)	14.0(7.6-34.9)	11.6(6.9-28.0)	5.1(2.7-10.3)	2.6(1.1-7.6)	12.6(5.6-28.2)	ns
**21**	12.2(5.6-25.4)	6.2(3.5-18.4)	3.1(1.2-7.9)	13.2(6.5-26.9)	12.5(6.4-19.5)	6.0(3.5-8.0)	2.6(1.4-9.8)	14.3(6.2-42.0)	ns
**34**	13.2(6.3-35.9)	7.3(3.5-16.8)	2.6(1.4-11.3)	15.1(5.7-32.0)	14.5(6.4-30.0)	6.4(3.2-11.0)	3.5(1.3-9.4)	13.4(7.0-40.9)	ns
**p-values**^**2**^	ns	ns	ns	ns	ns	ns	ns	ns	

Results are given as median and 10^th^- 90^th^ percentiles. The statistical methods used were Kruskal-Wallis^1^ comparing the two groups and Friedman’s ANOVA^2^ comparing the longitudinal process. ns = non-significant difference.

### Effects of intervention

#### ***Short-term effects***

In Tables 4 and 5, differences in total cortisol secretion, at screening (AUC-screening) and during the separate interventions (AUC-intervention) are shown.

**Table 4:** Area under the Curve (AUC) for short-term effects, before to 0 minutes after intervention

	***Group***	***AUC screening***^***1***^	***AUC intervention***^***2***^	**p-value**^**3**^
First intervention	RTM	553 (175-1118)	350 (143-1271)	0.249
	TM	571 (219-1226)	456 (189-954)	0.153
Eighth intervention	RTM	563 (188-1117)	338 (173-1030)	0.035*
	TM	525 (242-1215)	352 (221-656)	0.003*

1 AUC estimated according to individual intervention time for start and duration in minutes based on linear equation for daily AUC at screening. Median (10th and 90th perc).

2 AUC according to intervention duration and salivary concentration at start, 0 min after and 30 min after. Median (10th and 90th perc).

3 AUC intervention compared to AUC screening, Wilcoxon’s test for paired data.

*Statistically significant difference.

**Table 5:** **Area under the Curve (AUC) for short-term effects, before to *30 minutes *** **after Intervention**

	***Group***	***AUC screening***^***1***^	***AUC intervention***^***2***^	**p-value**^**3**^
First intervention	RTM	875 (292-1641)	582 (255-1939)	0.158
	TM	918 (337-1752)	662 (267-1366)	0.076
Eighth intervention	RTM	870 (315-1686)	562 (262-1614)	0.087
	TM	883 (373-1783)	491 (303-854)	0.004*

1 AUC estimated according to individual intervention time for start and duration in minutes based on linear equation for daily AUC at screening. Median (10th and 90th perc).

2 AUC according to intervention duration and salivary concentration at start, 0 min after and 30 min after. Median (10th and 90th perc).

3 AUC intervention compared to AUC screening, Wilcoxon’s test for paired data.

*Statistically significant difference.

After *the first* intervention, there was a significant decrease in salivary cortisol concentration in the TM group but not in total secretion (AUC) immediately after the intervention. In contrast, 30 min after the intervention, salivary cortisol concentrations were significantly decreased in both TM and RTM but the total cortisol secretion (AUC) was not changed in any group (Figure 3 and Tables 4 and 5).

After *the eighth* intervention, salivary cortisol concentrations were significantly decreased immediately and 30 min after intervention in both groups (Figure 3). Total salivary cortisol secretions (AUC) were significantly decreased immediately after the intervention in both groups but remained decreased only in the TM group (Tables 4 and 5).

Tables 4 + Table 5* Area under the Curve (AUC) for short-term effects, before to 0 minutes after intervention (4) and Area under the Curve (AUC) for short-term effects, before to 30 minutes after intervention (5).

We found no differences between groups in delta cortisol values and percentage changes after TM and RTM, as shown in Table 2. The immediate effects of intervention were not correlated to interventional time-point of the day (morning/afternoon), age, gender, BMI, or the duration of disease.

#### ***Long-term effects***

The diurnal cortisol concentrations are shown in Table 3. We compared the diurnal cortisol curve at baseline and during the study period (weeks 3, 8, 21, and 34). We found no change in diurnal rhythm or absolute cortisol concentrations during 26 weeks of follow-up. At baseline and after 3 weeks of the intervention period, the total cortisol secretion was higher during the night (8 pm–8 am) than day (8 am–8 pm) in both the TM group and the control group. Total cortisol secretion during the day was not significantly different between the TM and RTM groups.

#### ***Associations between salivary cortisol, clinical characteristics, and intervention***

No associations were found between salivary cortisol concentration and age, gender, weight, BMI, severity or duration of disease, interventional time-point of the day, or time-point or dose of levodopa intake.

## Discussion

In this study, we found an immediate effect (short-term effect) of TM on the hypothalamic-pituitary axis, with a significant decrease in cortisol concentration at both first and eighth time-points after TM. However, there were no significant differences between the TM and RTM groups at these time-points in the decrease in cortisol concentration. Nor were differences seen in cortisol concentrations between the groups due to the interventional time-point of the day. The delta cortisol and absolute values for salivary cortisol concentrations were similar in the two groups. The percentage decrease after intervention was more than 20% in both groups, which is in agreement with the results of another study in cancer patients receiving massage therapy [23]. To our surprise, we found no significant correlations between cortisol concentration and age, gender, weight, BMI, or disease duration. Our results further suggest that the diurnal pattern of cortisol secretion, i.e., the sensitivity of hypothalamic-pituitary axis function, was normal at baseline, before the intervention, and was unchanged during the interventions and up to 26 weeks after the last treatment session.

### Comparison of previous studies in this field

A recently published finding of the Parkitouch study was significantly increased morning salivary cortisol concentration compared with an age- and sex-matched healthy group [8]. In 2002, Hernandez-Reif et al [24] performed a pilot study on massage in PD patients, and to our knowledge, this is the first study of the effects of massage on HPA axis function in PD. They found no differences in urine cortisol secretion after the first and last interventions. However, in 2005 Field et al [25] showed changes in cortisol concentrations in saliva after massage therapy. This finding is in agreement with our results. A review article of massage therapy combined with analysis of cortisol in urine, saliva, or plasma in healthy and sick adults by Moraska et al [26] summarises the studies up to 2008. In eight studies, salivary cortisol was analysed at the first and/or sixth to 10th interventions. In 89% of studies, salivary cortisol concentration decreased after the first intervention. In four out of eight studies, salivary cortisol also decreased after the last treatment. This is in agreement with our results. Only one study has shown a significant decrease in urine cortisol after multiple treatments. No study has analysed diurnal or multiple salivary cortisol samples during intervention. During the passive non-interventional follow-up period, there was no previously published study that had analysed diurnal cortisol rhythm and/or multiple salivary cortisol samples.

### NMS

We did *not* include a self-reported mood questionnaire. Nor did we use a specific, sensitive questionnaire for depression and anxiety. However, our nurses and massage therapists met each person 10–16 times at these interventions. *No one* seemed to be depressed or very anxious, and no one reported signs of depression in the UPDRS I questionnaire. Instead, we used *specific questionnaires, Parkinson’s Disease Sleeping Scale (PDSS) and Short Form (36) Health Survey (SF36) Swedish version 1.0,* to compare sleep pattern and HRQoL. We found disturbed sleep and a low HRQoL, even lower than in patients who had had a major stroke 6 months earlier. These findings have recently been published [27].

### Interacting factors

Personal care and kind treatment are naturally of great value in all patient care. The placebo effect is also of essential importance, as described by Wormnes et al [28]. The TM method also included listening to tranquil and peaceful music. The volume was adjusted by the individual participant to a comfortable level to avoid stress and discomfort. In the pleasantly warm room, some smells of plants (aroma) were present but it was not a specific aromatherapy. To minimise and eliminate confounders because of these conditions, treatment of the control group (RTM) was identical in detail to that of the TM group, except for the specific moderate TM of the skin. The intention was to study the unique effect of this specific TM method and reduce the number of independent variables; therefore, we performed the study with two ‘active’ groups where only the presence of TM differed.

### Limitation of this study

Relatively few patients were included. The oldest PD patients were excluded due to difficulties in carrying out the extensive programme, and the risk of falling or balance problems in conjunction with the interventions on the massage table. However, compared with previous studies combining massage therapy and analysis of cortisol, our study is the second largest of nine controlled studies [26]. The distribution of participants between TM and RTM was somewhat distorted, 2:1, which was not our strategy at the start. However, as randomisation was performed with a computerised lottery technique and blinded, we had no influence on the distribution of patients to the groups. As the RTM group was quite small, it was possible that the spread of results within this group hid significant differences between TM and RTM (type II error). Therefore, a hypothetic comparison between the TM and RTM groups with a similar number of participants in each group (n = 28) was performed, using identical cortisol results as for the first 14 RTM participants. This theoretical model resulted in a significant difference between some factors representing pain and sleep, but not in the cortisol concentrations after interventions with the TM method compared with the RTM method (control). The study included no arm with a group given no intervention at all.

We did *not* include specific self-reported mood questionnaires after the interventions, but instead used a Parkinson’s disease-specific UPDRS I questionnaire [17]. A specific, sensitive questionnaire for depression and anxiety was not administered. It would be important to conduct further similar studies in bigger PD groups and/or other patients with chronic diseases and pain, to repeat and revaluate these positive short-term effects, before we can conclude the generalisability of the trial findings.

## Conclusion

Diurnal cortisol rhythm was normal in PD. Both TM and RTM interventions resulted in short-term significant decreases in salivary cortisol concentration and total secretion of cortisol during the day, with no significant difference between the groups. The effects on the HPA axis were dependent on the time-point of the intervention (first or eighth). A tendency towards a more pronounced decrease in cortisol concentration was seen when TM was added to the treatment. Cortisol concentrations at baseline and during the follow-up period were independent of age, gender, weight, BMI, and levodopa dose. The total diurnal cortisol secretion was lower during the day (8 am–8 pm) versus during the night (8 pm–8 am) at baseline. There was no recognisable long-term effect of the interventions on the HPA axis in terms of diurnal cortisol rhythm or total cortisol secretion.

## Abbreviations

AUC: Area under curve; BMI: Body mass index; CAM: Complementary and alternative methods; CAR: Cortisol Arousal Reaction; HRQoL: Health related quality of life; HPA-axis: Hypothalamic-pituitary-adrenal axis; PD: Parkinson’s disease; RTM: Rest to music; SF-36: The short form (36) health survey; TM: Tactile massage.

## Competing interests

The authors declare that they have no competing interests.

## Authors’ contributions

CJT, OS, AB, PAF, GH, MC, UL and HS were responsible for the study conception, data collection and design. BL, LR, LA, LA and PB performed the intervention. CJT and OS performed the data analysis. CJT, OS, PAF, GH and JL were responsible for drafting the manuscript. All authors read and approved the final manuscript.

## Pre-publication history

The pre-publication history for this paper can be accessed here:

http://www.biomedcentral.com/1472-6882/13/357/prepub

## Supplementary Material

Additional file 1 Short description of the Tactile Massage concept. Click here for file
