# Supplementary material for: Short- and long-term effects of tactile massage on salivary cortisol concentrations in Parkinson’s disease: a randomised controlled pilot study
Source: BMC Complement Altern Med. 2013 Dec 13;13:357. doi: 10.1186/1472-6882-13-357 (PMC3878723; doi:10.1186/1472-6882-13-357)
Supplement: Additional file 1 — Short description of the Tactile Massage concept. [file 1472-6882-13-357-S1.doc]

***Appendix I:* Short description of the Tactile Massage concept .**

The method “*Tactile Massage” -* a structured touch on the skin according to tactile methods.

*Tactile massage* means stroking with intention, using soft and encompassing hands on the skin. The strokes are given with respect, empathy and sensitivity for the special needs of the individual.

When performing *Tactile massage*, the **start** and **finish** are important. **Start** means that the therapist first places both hands on the body part to be massaged and briefly waits before beginning the strokes. **Finish** means the hands linger a short period before going on to the next body part. Except for the **start** and **finish**, the therapist’s hands maintain a steady flow from one stroke to another using a pressure that is comfortable for the receiver. It is this sequence of strokes, rhythm, flow and moderate pressure on the skin, developed through prior experience massaging Parkinson patients, which characterizes tactile touch.

The therapist: Conveys an attitude of dignity, respectfulness and caring for the individual’s integrity.
Room: Located in the hospital, approximately 22-24° C, lighted candles, a draped massage table and a chair.
Draping: With the exception of underwear, the recipient removes all their clothing, including jewellery, eye glasses and hair clips. A light blanket and pillows are used as desired by the receiver. Only the part to be stroked is uncovered.
The receiver: Is encouraged to close their eyes and avoid conversation not related to the massage.
Music: “Music for well being II” from “Fönix Music Wellness” is played at a sound level desired by the recipient.
Oil: Sufficient oil is used to facilitate this smooth flow of the hands. The entire *Tactile massage* is done with cold-pressed vegetable oil. The oil used in this study was “Fibro Olja” from Creacome AB and is a blend of sesame, rape, thistle, shea butter, arnica, olive and borage oils as well as Vitamin E and essential oils from ginger, lavender, rosemary and rose absolue.

On larger surface areas, i.e. back, thighs, chest, upper arm, etc, the entire palmer surface of the hands, including finger pads, is gently molded on and around the body part being massaged, following the contours of that body part. This type of stroking can be divided into two main categories:
1. Long strokes moving up or down the length of the body part. The fingers generally point toward the midline of the client’s body. Referred to as **effleurage**.
2. Strokes moving across the width of the body part. Referred to as **wringing**.

Where a more specific touch is desired, i.e. face, hands, feet and around the knees or elbows, the following strokes are used:
1. **Petrasage** **-**where the finger pads perform small, gentle circles.
2. **Spreading –** where the palmer surface of the fingers or thumb stroke from the midline to the sides of the body part.

For the first visit, the therapist describes a full body massage and the recipient’s questions are answered.

Experience has shown that most Parkinson patients prefer to start the massage lying face down (prone) on the table. The therapist always asks the recipient if it feels OK to start the massage.

Sequence for *Tactile massage*

1. Back of legs

2. The back

3. Back of the head

The recipient turns over

4. Abdomen

5. Chest

6. Face, ears

7. Front of head

8. Right arm, then right hand

9. Left arm, then left hand

10. Left leg, then left foot

11. Right leg, then right foot

All strokes are performed twice.

*Tactile massage* is ended by the therapist holding onto both feet outside the cover then gently releasing the feet.

The therapist now releases contact with the recipient, who is invited to lie still for a moment before water, which always is nearby, is offered.

The therapist thanks the recipient for the privilege of massaging and assists them off the table and with dressing, if the recipient so desires.

Each session is concluded with a discussion of what the recipient has felt and experienced.
